# Outcome Analysis of Total Hip Arthroplasty in the Setting of Post-traumatic Arthritis of the Hip: A Case Report

**DOI:** 10.7759/cureus.54053

**Published:** 2024-02-12

**Authors:** Abhishek Chaudhary, Gajanan Pisulkar, Shounak Taywade, Abhiram A Awasthi, Ankur Salwan

**Affiliations:** 1 Orthopaedics, Jawaharlal Nehru Medical College, Datta Meghe Institute of Higher Education and Research, Wardha, IND

**Keywords:** case report, orthopedics, implant removal, total hip arthroplasty, post-traumatic arthritis

## Abstract

This case report outlines the successful management of post-traumatic arthritis (PTA) in the left hip of a 60-year-old male with a history of a subtrochanteric femur fracture treated with Jewett Nail Plate osteosynthesis four decades ago. Despite seeking relief from various healthcare facilities and attempting alternative therapies, the patient experienced persistent pain and limited mobility. The decision was made to perform elective implant removal followed by total hip arthroplasty (THA). The surgical intervention involved a modified posterior approach, addressing specific challenges such as acetabular superior wall deficit and femoral sclerosis. A comprehensive management approach, considering the patient's complex medical history, including prolonged tobacco use and alcohol consumption, contributed to the successful outcome. Postoperative care included a multimodal drug cocktail for pain management and a well-coordinated physiotherapy program. Postoperative imaging confirmed the procedure's success, and the patient exhibited significant improvement in pain relief and functional outcomes. This case underscores the importance of a tailored and comprehensive approach in managing PTA, showcasing the effectiveness of elective implant removal followed by THA in addressing PTA of the hip.

## Introduction

Post-traumatic arthritis (PTA) of the hip is a degenerative joint condition resulting from previous trauma to the hip joint, such as fractures or dislocations. This condition can lead to chronic pain, functional impairment, and decreased quality of life. The long-term sequelae of hip fractures, especially those treated with internal fixation devices, may predispose individuals to the development of PTA over time [1]. The case presented here exemplifies the challenges and successful management of a patient with PTA of the left hip, stemming from a subtrochanteric femur fracture treated with Jewett Nail Plate osteosynthesis four decades ago. The prevalence of PTA following hip fractures is well documented, with studies indicating that up to 10-15% of patients may develop arthritis within 10-15 years post-injury [2]. The mechanisms underlying the development of PTA are multifactorial and include joint incongruity, altered biomechanics, and post-traumatic soft tissue damage [3]. In this case, the patient's history of hip trauma and the use of internal fixation served as predisposing factors for the subsequent development of PTA.

Despite the availability of various treatment options, including pharmacotherapy, physiotherapy, and alternative medicine, surgical intervention often becomes necessary for advanced cases of PTA to alleviate pain and restore joint function [4]. Total hip arthroplasty (THA) has proven to be an effective and widely accepted surgical intervention for end-stage hip arthritis, providing patients with significant pain relief and improved functional outcomes [5]. In this case, the decision to perform THA followed the elective removal of the existing implant, addressing the underlying cause of PTA and providing a durable solution for joint reconstruction. It is essential to consider patient-specific factors, such as age, comorbidities, and lifestyle, when planning and executing surgical interventions for PTA. The presented case involves a 60-year-old male with a history of prolonged tobacco use, alcohol consumption, and prior hip surgery. The influence of these factors on surgical outcomes and complications necessitates a tailored and comprehensive management plan. The literature emphasizes the importance of careful preoperative planning and meticulous surgical technique in achieving optimal results in patients undergoing THA for PTA [6]. Additionally, managing the patient's postoperative course involves a multidisciplinary approach, integrating pain management strategies, physiotherapy, and close monitoring for potential complications.

This case report contributes to the existing body of knowledge on managing PTA, showcasing the successful integration of implant removal and subsequent THA in a patient with a complex medical history. By highlighting the challenges faced and strategies employed, this case underscores the importance of a comprehensive approach to address the multifaceted nature of PTA of the hip.

## Case presentation

A 60-year-old male has been experiencing pain and limited movement in his left hip since 2022. The patient, who underwent surgery for a left subtrochanteric femur fracture 40 years ago with an implanted device still in place, reported a sudden onset of pain in the left hip that progressed gradually. Seeking relief, he initially visited a private hospital in central India the next day, where prescribed medications provided temporary relief. The pain worsened with movement, particularly at night, but improved with rest and medication. After a month, the patient sought treatment at an Ayurvedic hospital, where he received drugs and therapy offering limited pain relief for a short duration. Subsequently, on November 14, 2022, the patient visited a rural hospital in central India for an X-ray and medication, temporarily alleviating the pain. Seeking further assistance, he visited a government hospital in a metro city on November 15, 2022, where prescribed medications provided minimal relief for a brief period.

**Figure 1:**
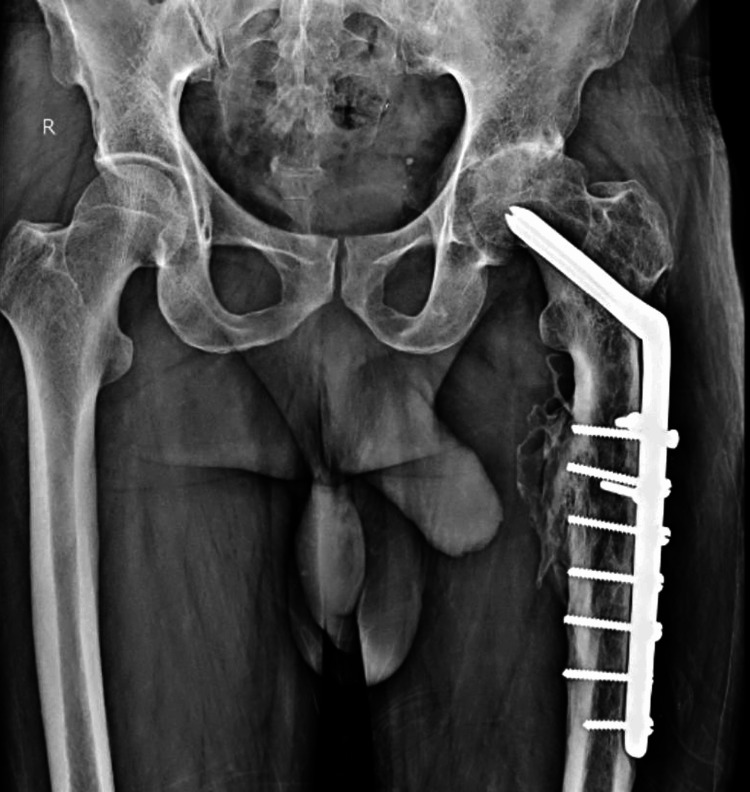
X-ray of the united subtrochanteric femur fracture on the left side with the implant in situ.

The patient, who underwent left hip surgery with Jewett Nail Plate osteosynthesis 40 years ago (Figure 1), has a history of alcohol consumption, daily tobacco chewing, and smoking for 50 years (twice a month, daily, and twice per week, respectively).

The management plan involved the elective removal of the implant from the left hip on November 29, 2022 under spinal anesthesia. The patient was positioned laterally during the procedure, and the implant was successfully removed without complications. Subsequently, the patient was scheduled for THA on the left side.

**Figure 2:**
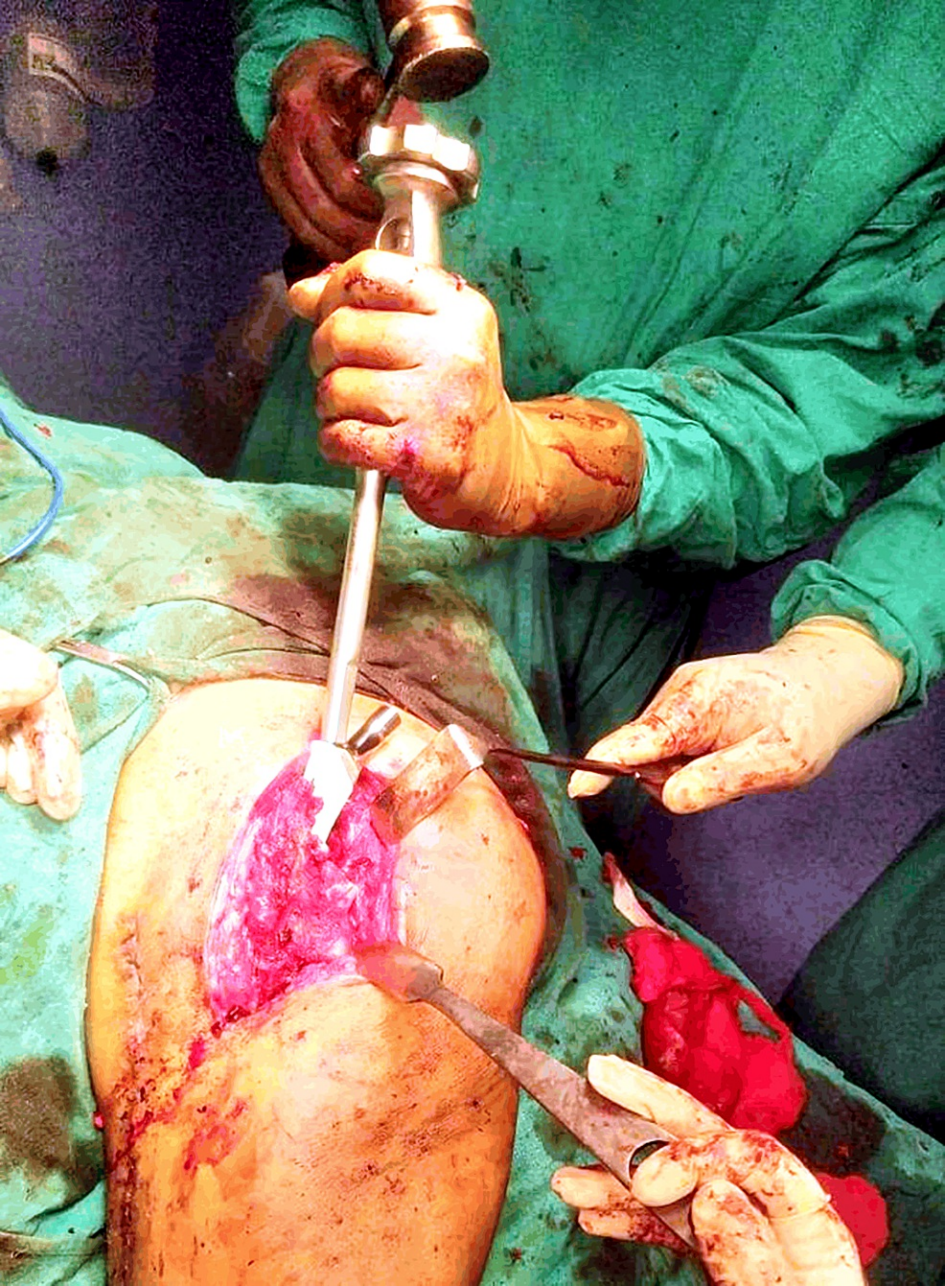
The impaction of the femoral stem into the femoral canal.

Under spinal anesthesia, the patient was positioned laterally, and a modified posterior approach was used. A meticulous incision of 8 cm was made, followed by soft tissue dissection, revealing significant fibrosis with no dissection of external rotators. The procedure included capsule and neck cuts, head extraction, and identification of an acetabular superior wall deficit. Acetabular serial reaming, femoral canal preparation, and a size 1 femoral stem implantation were performed. A 52 mm cup with two screws and an oblique liner was utilized. Femoral sclerosis was addressed through reaming with K-wires, drills, and seamers (Figure 2).

**Figure 3:**
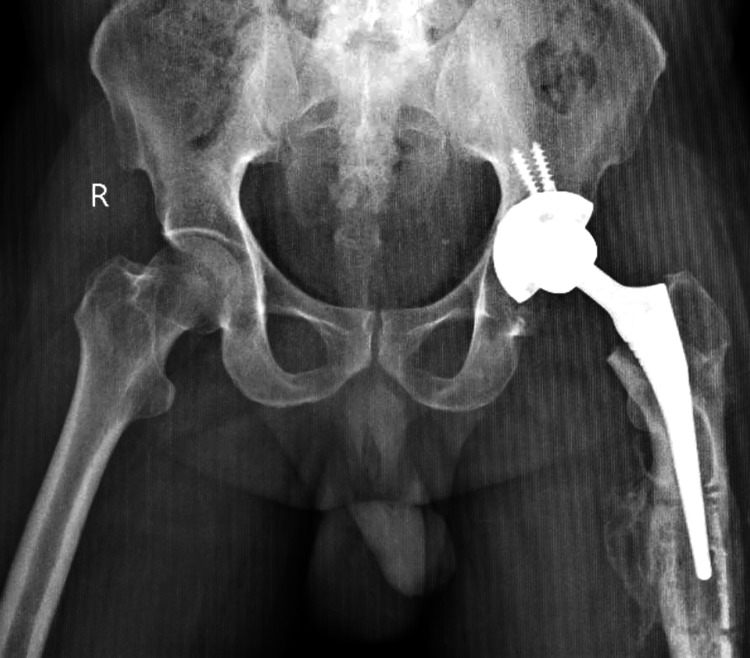
X-ray of the operated case of total hip replacement on the left side with the implant in situ.

Post-implantation, a multimodal drug cocktail was administered, and the surgical site was closed. Negative suction drainage was implemented, and the wound was closed with Vicryl and staples. The patient was moved to the ortho recovery room, where postoperative physiotherapy commenced. Suture removal on postoperative day 12 revealed a healthy suture site. A subsequent follow-up X-ray confirmed the success of the procedure after three months (Figure 3).

## Discussion

The presented case highlights the successful management of PTA in the hip through a comprehensive approach involving elective implant removal and subsequent THA. The patient's history of a subtrochanteric femur fracture treated with Jewett Nail Plate osteosynthesis 40 years ago, combined with a complex medical background, posed unique challenges that were effectively addressed through a tailored treatment plan. The development of PTA after hip trauma, especially in cases involving internal fixation, is a recognized phenomenon [7]. Studies have reported that patients with hip fractures treated with internal fixation may develop arthritis over time due to factors such as altered joint biomechanics and post-traumatic soft tissue damage [8]. In this case, the decision to proceed with elective implant removal followed by THA aimed to address the underlying cause of PTA and provide a durable solution for joint reconstruction. The patient's history of prolonged tobacco use, alcohol consumption, and prior hip surgery added complexity to the management strategy. These factors influence surgical outcomes, postoperative complications, and patient recovery [9,10]. Despite the challenges associated with the patient's lifestyle, the successful outcome observed in this case emphasizes the importance of careful preoperative planning, patient counseling, and a multidisciplinary approach to optimize results.

THA remains the gold standard for end-stage hip arthritis, providing significant pain relief and functional improvement [11]. The surgical procedure involved a modified posterior approach, meticulous soft tissue dissection, and addressing specific challenges such as acetabular superior wall deficit and femoral sclerosis. The utilization of a multimodal drug cocktail for postoperative pain management, negative suction drainage, and a well-coordinated physiotherapy program contributed to the patient's overall positive outcome. The success of the surgical intervention was confirmed by postoperative imaging, indicating proper implant placement and joint stability. Suture removal on postoperative day 12 revealed a healthy wound site, highlighting the effective wound closure and postoperative care protocols. While the presented case demonstrates favorable outcomes, it is crucial to acknowledge potential limitations. The long-term success and durability of the THA must be monitored through extended follow-up, considering factors such as implant wear, osteolysis, and potential complications associated with the patient's lifestyle.

## Conclusions

In conclusion, the comprehensive management of PTA in the left hip, as presented in this case, has demonstrated the efficacy of elective implant removal followed by THA. The decision to perform THA after elective implant removal effectively addressed the underlying cause of PTA, providing a durable solution for joint reconstruction. Despite the patient's complex medical history, including prolonged tobacco use, alcohol consumption, and prior hip surgery, the meticulous surgical intervention, utilizing a modified posterior approach, contributed to positive short-term outcomes. The success of the procedure was confirmed through postoperative imaging, and the patient exhibited substantial improvement in pain relief and functional outcomes. While these results are promising, long-term follow-up is essential to assess the durability and sustained success of the THA, and continued monitoring will be crucial for the early identification and management of any potential complications. This case emphasizes the importance of a tailored and multidisciplinary approach in achieving optimal outcomes for patients with PTA, offering valuable insights for future considerations in the management of PTA of the hip.
